# 4-hydroxysesamin protects rat with right ventricular failure due to pulmonary hypertension by inhibiting JNK/p38 MAPK signaling

**DOI:** 10.18632/aging.205808

**Published:** 2024-05-08

**Authors:** Lingnan Zhang, Xinshun Gu

**Affiliations:** 1 Department of Cardiovascular, Affiliated Hospital of Hebei University, Baoding 071000, Hebei, China; 2 Department of Cardiovascular, The Second Hospital of Hebei Medicine University, Shijiazhuang 050000, Hebei, China; 3 Department of Cardiovascular Sciences, Hebei Medical University, Shijiazhuang 050017, Hebei, China

**Keywords:** pulmonary hypertension, 4-hydroxysesamin, right ventricular failure, JNK/p38 MAPK signaling pathway

## Abstract

The specific mechanism of 4-hydroxysesamin (4-HS), a modification of Sesamin, on right ventricular failure due to pulmonary hypertension (PH) is ominous. By creating a rat model of PH *in vivo* and a model of pulmonary artery smooth muscle cell (PASMC) hypoxia and inflammation *in vitro*, the current work aimed to investigate in depth the molecular mechanism of the protective effect of 4-HS. In an *in vitro* model of hypoxia PASMC, changes in cell proliferation and inflammatory factors were detected after treatment with 4-HS, followed by changes in the JNK/p38 MAPK signaling pathway as detected by Western blot signaling pathway. The findings demonstrated that 4-HS was able to minimize PASMC cell death, block the JNK/p38 MAPK signaling pathway, and resist the promoting effect of hypoxia on PASMC cell proliferation. Following that, we found that 4-HS could both mitigate the right ventricular damage brought on by MCT and had a protective impact on rats Monocrotaline (MCT)-induced PH in *in vivo* investigations. The key finding of this study is that 4-HS may protect against PH by inhibiting the JNK/p38 MAPK signaling pathway.

## INTRODUCTION

PH is a prevalent cardiovascular condition, and right ventricular failure is one of the main prevalent complications of PH and one of the leading causes of death in patients. It is characterized by a persistent elevation of pressure in the right ventricle, which results from changes in the myocardium and cardiac structure caused by chronic PH [1, 2]. In recent years, some important advances have been made as the study of PH and right ventricular failure has intensified. Studies have shown that the development and progression of PH is related to a variety of factors, including heredity, smoking, hypertension, obesity, hyperlipidemia, diabetes, and heart disease [3, 4]. These factors can lead to pulmonary artery disease as well as changes in right ventricular muscle and cardiac structure, which can cause right ventricular failure [5].

With advances in medicine, progress has been made in the treatment of PH and right ventricular failure. Pharmacologic therapy is the most common treatment, including the use of antihypertensive drugs, diuretics, ACE inhibitors, β-blockers, and ARNIs [6]. Interventional and surgical treatments can also improve right ventricular function and enhance patient quality of life [4]. The management of right ventricular failure and PH still faces numerous difficulties. For instance, many patients require long-term treatment with drugs that can cause serious side effects. In addition, surgical treatment requires great skill and precision to ensure patient safety and improve outcomes [7]. Therefore, there is a need to find novel low-toxicity and efficient targeted drugs to provide new treatment options for patients with current clinical PH.

Sesamin is a polyphenolic compound containing several hydroxyl and methoxy functional groups. It is mainly derived from the seeds and roots of plants such as sesame seeds, black sesame seeds, etc. [8]. Sesamin has a wide range of biological actions that can both prevent and treat various diseases, such as protecting cells from oxidative damage and having a strong antioxidant capacity [9]. It can reduce cyclophosphamide-induced nephrotoxicity, reduce inflammatory response, relieve pain and swelling [10]. Sesamin also has anticancer properties [11, 12]. Although Sesamin has demonstrated great potential in the treatment of many diseases, low bioavailability and limited activity levels [13] have limited clinical development. 4-HS are laboratory synthesized modifications of Sesamin, and the pharmacological activity of 4-HS has not yet been extensively studied. Therefore, the aim of this preclinical study was to investigate the protective role of 4-HS in right ventricular failure due to PH.

## MATERIALS AND METHODS

### Cell culture

Pulmonary artery smooth muscle cells (PASMC) were purchased from the Cell Bank of the Chinese Academy of Sciences, Shanghai, China. DMEM high glucose medium (11995, Gibco, USA) containing 10% fetal bovine serum (YSN0121, ExCell Bio, China) and 1% penicillin-streptomycin mixture (P1400, Solarbio, China) was configured, and the cells were cultured in the configured DMEM medium and placed in an incubator at 37°C with 5% CO_2_ (CI-191C, Jiemei Electronics, China). The experimental agent 4-HS was provided by Professor S. Zhang (State Key Laboratory of Bioactive Substances and Functions of Natural Medicines, Institute of Materia Medica, Chinese Academy of Medical Sciences and Peking Union Medical College, Beijing, China). MCT (HY-N0750), Sesamin (HY-N0121) and Anisomycin (HY-18982) were purchased from Med Chem Express Shanghai, China.

### *In vitro* construction of a cellular model of PH

PASMC were pretreated with different drugs (4-HS, Sesamin, and Anisomycin) for 24 h. LPS added in the pulmonary arterial hypertension model group to give a final concentration of 1 μg/mL was placed in a cell culture incubator at 1% O_2_, 37°C for 8 h. At the end of the incubation, they were used for subsequent experimental manipulations.

### CCK8

PASMC in good growth condition were taken, washed three times with sterile PBS, and the cells were incubated in a culture incubator (P6730, Solarbio) for 2 min after the addition of appropriate amount of trypsin, PASMC were taken out, and the digestion was terminated by the addition of DMEM complete medium, centrifugation was carried out at 1200 rpm for 3 min, the cells were resuspended by adding the appropriate amount of medium, and the cells were cultured at 3000 cells per well. Cells were added to 96-well plates, incubated overnight, and different drugs were added to pretreat the cells for 24 h. Pulmonary arterial hypertension was modeled as previously described, and 10 μL of CCK8 (CA1210, Solarbio) solution was added to each well after 24 h of incubation in an incubator, and the absorbance at 450 nm was measured by an enzyme marker (Flash, ReadMax 1200) after 3 h of incubation.

### EDU

As described, the cells were inoculated in six-well plates with 10 μmol EDU solution (C0075S, Beyotime, China) added to each well and incubated in a 5% CO_2_ incubator at 37°C for 2 h. After incubation, the medium was removed and 1 mL fixing solution (P0099, Beyotime) was added to each well for incubation at room temperature for 15 min. Remove fixing solution, add 0.3% Triton X-100 and incubate at room temperature for 15 min. The cells were washed three times with PBS and incubated at room temperature for 5 min with DAPI dye (C1002, Beyotime). After incubation, the images were observed by inverted fluorescence microscope (ICX41, Sunny Optical Technology Co., Ltd., China), and stored for subsequent statistical analysis of ImageJ 1.52a.

### ELISA

Take 50 μL of pre-treated rat serum and cell supernatant, add to ELISA microtiter wells and incubate for 1 h, then react with HRP for 30 min, after three washes with PBS for 5 min each time, and add the substrate TMB to develop the color for 15 min. TMB is converted to blue color catalyzed by the HRP enzyme, and converted to the final yellow color in the presence of acid. The absorbance (OD value) was measured at 450 nm with an enzyme marker and the concentration was calculated by standard curve. IL-6 ELISA KIT (Solarbio, SEKH-0013 and SKER-0005), IL-10 ELISA KIT (Solarbio, SEKH-0018 and SKER-0006), IL-1β ELISA KIT (Solarbio, SEKH-0002 and SKER-0002), TNF-α ELISA KIT (Solarbio, SEKH-0047 and SKER-0009).

### Western blot

PASMC cell samples and tissue samples were collected from each experimental group, added with tissue cell lysate and placed on ice for 25 min (the tissues need to be grinded with a homogenizer in advance), and centrifuged at 12,000 rpm for 10 min at 4°C. The protein concentration was measured according to the instructions of BCA Protein Quantification Kit (Solarbio, PC0020), and according to the protein concentration, add the corresponding 5× protein upload buffer (Solarbio, P1040) and supplemented with tissue cell lysate, and the protein samples were heated for 10 min in a water bath at 95°C, and then put into a refrigerator at −80°C for storage. For gel electrophoresis, the voltage was adjusted to 80 V, and after 35 min, the voltage was modulated to 120 V. After waiting for another 60 min, the electrophoresis was ended. Modulate to a constant current of 260 mA, 1 h to end the membrane transfer, take out the PVDF membrane, put the PVDF membrane in 5% skimmed milk powder closure for 2 h. After the closure, wash it with 1 × TBST solution for 3 times, each time for 30 min, and incubate the corresponding primary antibody placed at 4°C overnight. Remove the PVDF membrane, wash it with 1 × TBST solution 3 times, 30 min each time, prepare the secondary antibody with 5% skimmed milk powder, incubate for 1 h. At the end of the incubation, wash it with 1 × TBST solution 3 times, 30 min each time, dropwise add the chemical developing solution, image it in the gel imager, and the picture was used in the Image J 1.52a for subsequent data processing and analysis. JNK (Affinity Biosciences Ltd., USA, #AF6318), p-JNK (Affinity Biosciences Ltd., #AF3318), p38 MAPK (Abcam, UK, ab308333), p-p38 MAPK (Affinity Biosciences Ltd., #AF4001), GAPDH (Affinity Biosciences Ltd., #AF0911), Goat Anti-Rabbit IgG (H+L) HRP (Affinity Biosciences Ltd., #S0001), Goat Anti-Mouse IgG (H+L) HRP (Affinity Biosciences Ltd., #S0002), CDK2 (HUABIO, USA, ET1602-6), CyclinD1 (HUABIO, ET1601-31), PCNA (Abcam, ab29).

### TUNEL staining

Paraffin sections of rat heart tissue were deparaffinized in distilled water, washed 3 times with PBS for 5 min, and proteinase K working solution was washed 3 times with PBS for 5 min at 37°C for 30 min, and H_2_O_2_ was washed 3 times with PBS for 5 min at room temperature for 10 min. TdT enzyme reaction solution was washed with PBS for 5 min at 37°C for 60 min, protected from light. Streptavidin-HRP solution at 37°C for 30 min, protected from light, washed 3 times with PBS for 5 min each time, DAB for color development, washed 3 times with PBS for 5 min each time, hematoxylin re-staining and sealing, and bright field panoramic scanning.

### Sirius red staining

Dewaxing to water: xylene I 5 min, xylene II 5 min, xylene III 5 min, anhydrous ethanol 1 min, 95% ethanol 1 min, 75% ethanol 1 min, distilled water wash for 5 min. Dropwise addition of ferric hematoxylin staining solution for 5–10 min, distilled water wash for 10–20 s to wash away excess staining solution. The sections were washed with tap water for 5 min, and stained with Sirius red stain for 15–30 min, and rinsed slightly under running water to remove the staining solution from the surface of the sections. Dehydration and transparency: 75% ethanol for 1 min, 95% ethanol for 1 min, anhydrous ethanol for 1 min, xylene for 3 min, 2 min each time, and neutral gum sealing.

### Cardiopulmonary function indicator test

Preparation of rat pulmonary artery catheter: take the length of about 15 cm PV-1 tube, form a radius of about 3 mm smooth arc-shaped cannula preparation, marking at 4 cm from the end. Carotid artery and pulmonary artery cannulation: Pentobarbital sodium (Merck, CAS: 57-33-0, Germany) 45 mg/kg anesthesia rats, supine position fixed on the surgical plate, incision of the neck skin, blunt separation of subcutaneous tissues and muscle layer, stripping of the carotid artery and the right external jugular vein, the pressure transducer is connected to the heparin-filled saline PE-50 tube inserted into the carotid artery, the pressure transducer is connected to the polysystemic physiological recorder through the carrier amplifier, the pressure recorded represents the systemic circulatory blood pressure. The pulmonary artery cannula prepared as described above was filled with heparin saline and inserted into the right external jugular vein. As soon as the pressure baseline rose and a pulmonary artery waveform appeared, the PV-1 catheter was secured and tracing of ppa was initiated. Fulton’s index of right ventricular hypertrophy (RVH) was assessed as the ratio of right ventricular weight to left ventricular (LV) and septal (S) weights (right ventricle/(LV+S) ratio).

### Cardiac ultrasound

For echocardiography, the animals were anesthetized under gas anesthesia (isoflurane-vifluorane, Virbac 1.8-2% in a 1:1 oxygen:air mixture). Hair was removed from the thoracic area (depilatory cream was used for sensitive skin), and the animals were placed on a heated platform connected to an ultrasound system (Vevo^®^ 3100 LT, 69 Fujifilm VisualSonics Inc., Toronto, Canada) to record ECG and respiratory rate. Images were acquired using a 70 MS-550D (55 MHz) transducer to determine the AT:ET ratio and CO; this transducer is specifically designed for cardiac imaging in rat (VisualSonics). Hemodynamic parameters were measured in nonventilated anesthetized rat using a closed-chest technique by introducing a 1.4 f Miller catheter (ADInstruments, Paris, France) into the jugular vein and directing it to the right ventricle, inhaling 2.0% isoflurane on room air. After hemodynamic assessment was completed, the heart and lungs were removed en bloc to assess changes in the right ventricle and pulmonary vasculature.

### *In vivo* experiment

4–6 weeks old SD rats, male, weighing 150–200 g, were housed in the Affiliated Hospital of Hebei University, given sufficient sterile feed and sterile water, and formal experiments were started after 7 days of feeding, and were randomly divided into 4 groups (*n* = 10), the pulmonary arterial hypertension modeling group was injected subcutaneously in the back of the SD rats with 70 mg/kg of MCT, the control group was injected with the same dose of saline, the 4-HS-treated group was injected intraperitoneally with 20 mg/kg of 4-HS the day before the MCT injection, and then 4-HS was injected intraperitoneally every 3 days, 6 weeks later, because some rats in the MCT-induced PH group died, five rats from each group of surviving rats were randomly selected for the subsequent experiments, and the cardiac ultrasound of rats was detected using an animal ultrasound system to detect cardiorespiratory function indexes of rats, and cardiopulmonary function indexes were taken. Rats’ cardiopulmonary tissues and pulmonary arteries for subsequent experiments (*n* = 5).

### Statistical analysis

All data were statistically analyzed using SPSS software. Measured data were expressed as mean ± standard deviation (SD). Differences between groups were verified by one-way or two-way analysis of variance (ANOVA), and differences between two groups of data were analyzed by *t*-test, and all experimental data were repeated more than three times, and the *P*-value < 0.05 was statistically significant.

### Availability of data and materials

Data will be made available on request.

## RESULTS

### 4-HS protects against LPS+ hypoxia-induced PASMC cell injury

**Figure 1:**
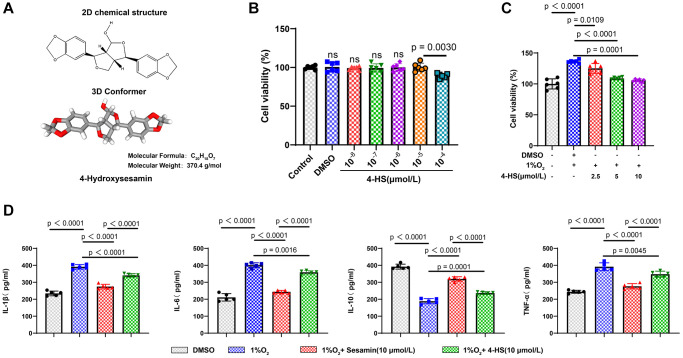
**Effect of Sesamin and 4-HS on the inflammatory response to PASMC cell viability.** (**A**) 2D and 3D molecular structural formulae of 4-HS and the relative molecular mass of 4-HS. (**B**) CCK8 assay to detect the effect of different concentrations of 4-HS on cellular value-added after acting on PASMC for 24 h. (**C**) CCK8 assay to detect the effect of 4-HS on the cell viability of PASMC in inflammatory and hypoxic environments. (**D**) ELISA assay to detect changes in expression of inflammatory factors IL-1β, IL-6, IL-10, and TNF-α in inflammatory and hypoxic environments under 4-HS expression changes of inflammatory factors IL-1β, IL-6, IL-10, and TNF-α after 24 h of 4-HS treatment.

Figure 1A Show the 2D and 3D molecular constructs and relative molecular mass of 4-HS. We first evaluated the effect of 4-HS on PASMC cell viability, aiming to find the nontoxic concentration of 4-HS on PASMC, CCK8 results showed that 4-HS at 100 μmol/L had an inhibitory effect on cellular PASMC cell viability (*P* = 0.0020) as shown in Figure 1B. Therefore, we chose 10 μmol/L as the main drug for the subsequent experiment’s concentration.

Subsequently, we established a cellular model of hypoxia-induced environment in PASMC to simulate PH *in vivo*, and the CCK8 results showed that cell viability was significantly enhanced after hypoxia induction compared with the control group (*P* < 0.0001), whereas the group pre-treated with 4-HS for 24 h had decreased cell viability compared with that of the hypoxia induction group, and in a concentration-dependent manner (Figure 1C). To confirm the stronger anti-inflammatory effect of 4-HS than Sesamin, we detected the changes of inflammatory factors by ELISA assay under the environment of hypoxia induction in PASMC, and the results showed that the 4-HS and Sesamin groups were able to significantly down-regulate the expression of IL-6, IL-1β, and TNF-α, and significantly up-regulate the expression of IL-10 compared to hypoxia-induced group, but the 4-HS group had stronger anti-inflammatory efficacy compared with Sesamin group (Figure 1D).

### The protective effect of 4-HS may act via inhibition of JNK/p38 MAPK signaling homology

**Figure 2:**
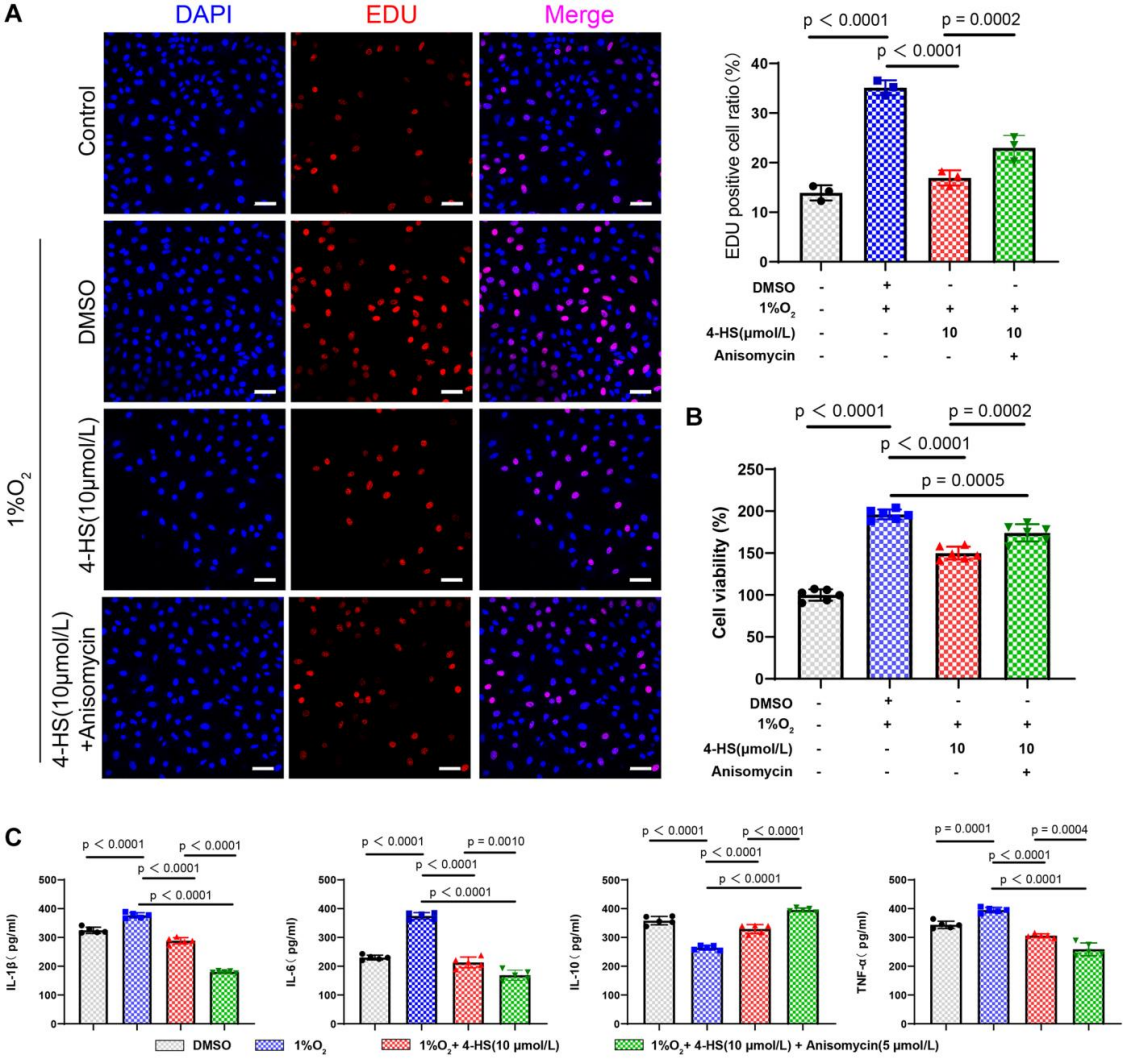
**4-HS protects PASMC in inflammatory stimuli and hypoxic environments.** (**A**) EDU staining to detect changes in the proliferative capacity of treated PASMC (Scale bar, 50 μm). (**B**) CCK8 assay to detect changes in the proliferative capacity of PASMC after treatment with 4-HS PASMC treated with 4-HS and Anisomycin for 24 h. (**C**) ELISA assay to detect changes in the expression of inflammatory factors IL-1β, IL-6, IL-10, and TNF-α in PASMC treated with 4-HS in combination with Anisomycin for 24 h in LPS and hypoxia-stimulated environments.

We verified whether the protective effect of 4-HS on the proliferation of PASMC was also achieved by regulating the JNK/p38 MAPK signaling pathway by CCK8 (Figure 2B) and EDU (Figure 2A) staining experiments. The results showed that 4-HS pretreatment was able to alleviate the promoting effect of hypoxia-induced on PASMC cell proliferation (*P* < 0.0001), whereas the Anisomycin group showed a significant enhanced in the cell proliferation ability compared with the 4-HS pretreatment group (*P* = 0.0002), after which we evaluated whether the anti-inflammatory effect of 4-HS is also associated with the JNK/p38 MAPK signaling pathway. The results showed that the levels of IL-6, TNF-α, and IL-1β were up-regulated and the expression level of IL-10 was down-regulated in the Anisomycin group compared with the 4-HS pretreatment group as shown in Figure 2C.

**Figure 3:**
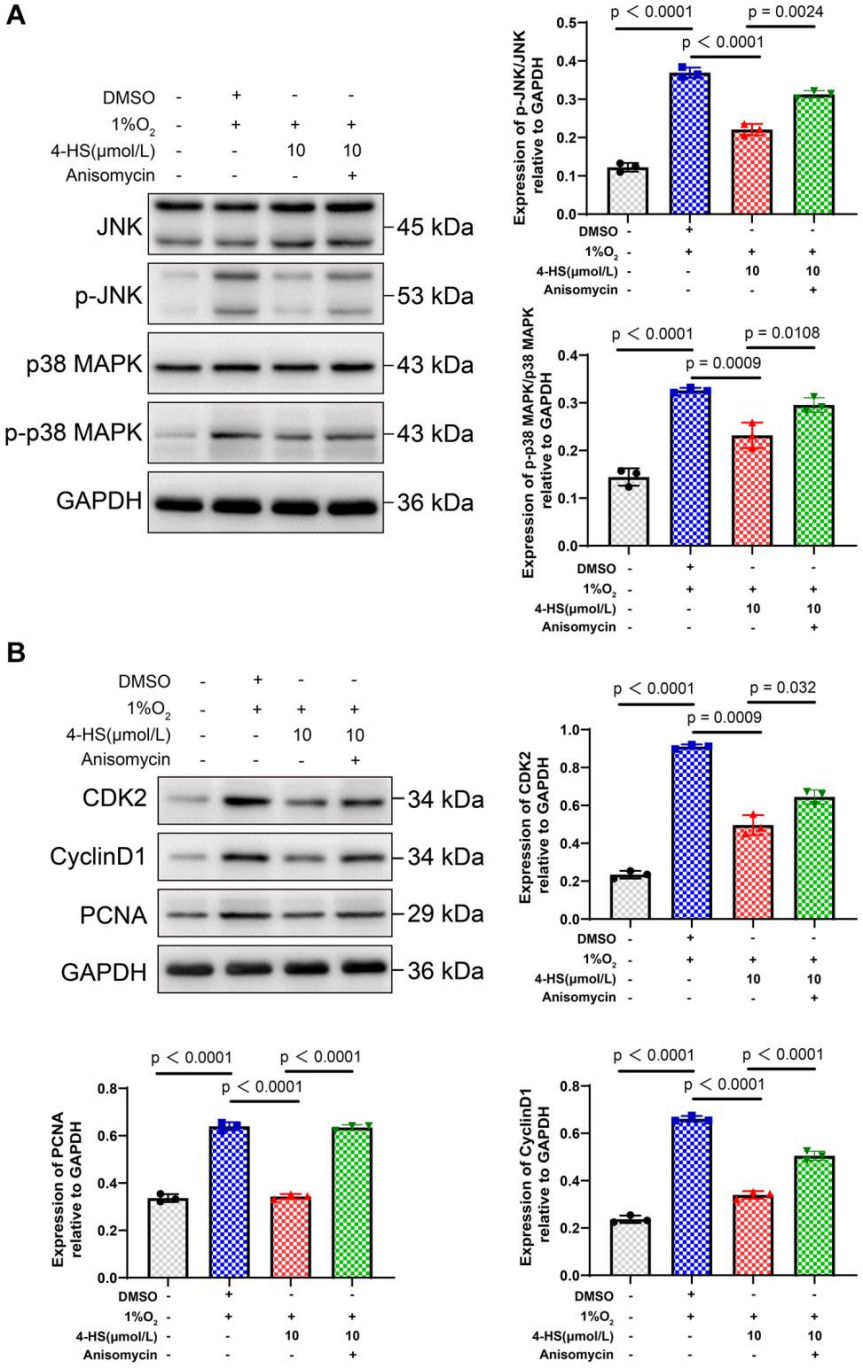
**4-HS expression of JNK/MANK signaling pathway and proliferation-related proteins.** (**A**) Western blot assay to detect the changes in the expression of JNK, p-JNK, p38 MAPK, and p-p38MAPK proteins in PASMC after treatment. (**B**) Western blot assay to detect the changes in the expression of CDK2, CyclinD1, and PCNA proteins in PASMC after treatment.

Anisomycin is the activator of the JNK signaling pathway. In order to verify the effect of 4-HS on the JNK/p38 MAPK signaling pathway, we tested the expression of related proteins by Western blot assay. The results showed that the hypoxia group had no effect on the expression of JNK and p38 MAPK proteins. However, the expression levels of P-JNK (*p* < 0.0001), P-P38 MAPK (*P* < 0.0001), CDK2, CyclinD1 and PCMA were enhanced in hypoxia-induced group. The expression of P-JNK (*p* < 0.0001), P-P38 MAPK (*P* < 0.0001), CDK2, CyclinD1 and PCMA were inhibited in the 4-HS group compared with the hypoxia-induced group. The addition of Anisomycin was able to counteract the inhibition of 4-HS on related proteins. This suggests that 4-HS can inhibit PASMC proliferation induced by hypoxia by inhibiting JNK/p-38MAPK signaling pathway (Figure 3A, 3B).

### 4-HS protects against pulmonary arterial hypertension in rats

**Figure 4:**
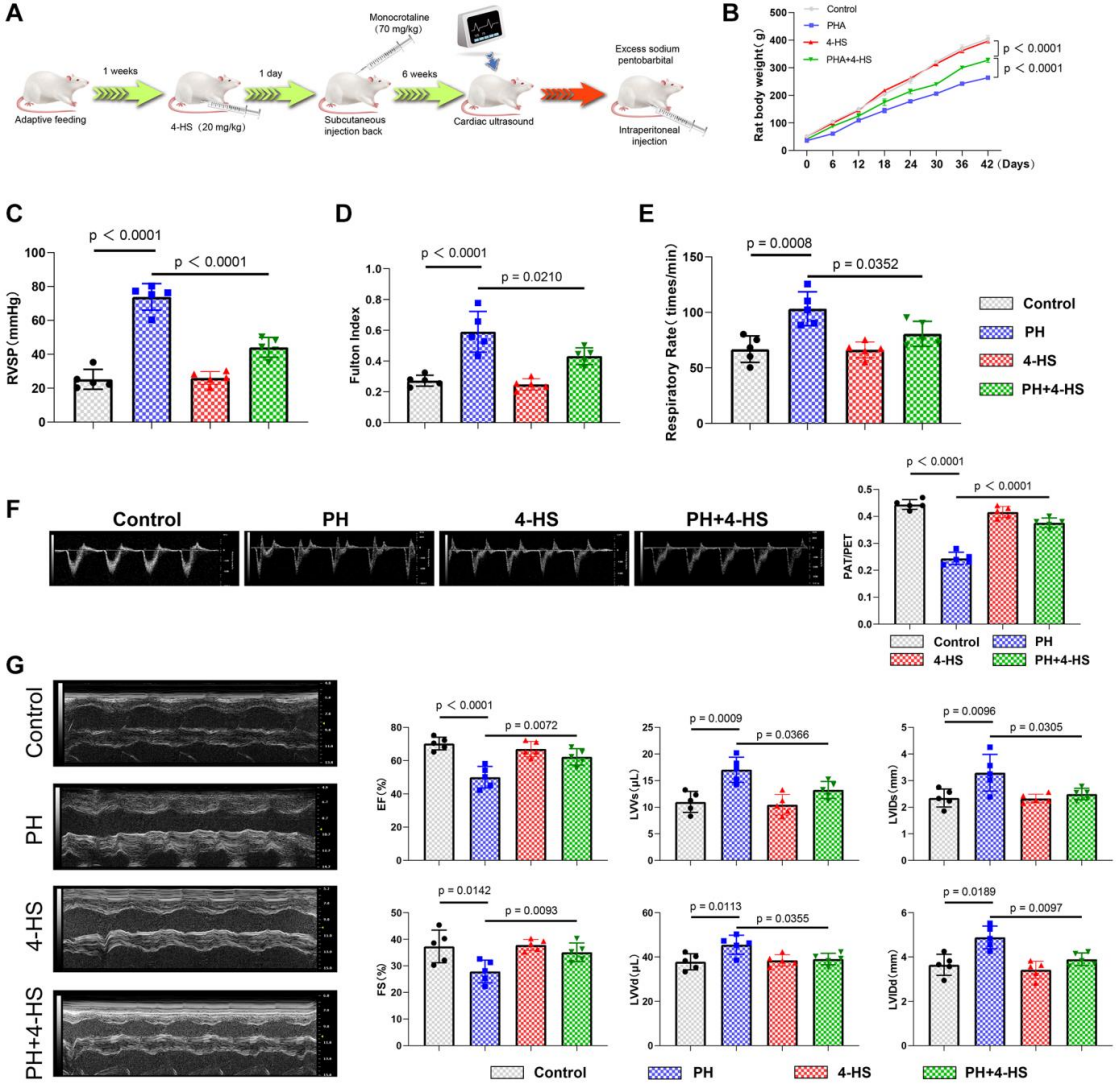
**Protective effect of 4-HS on cardiopulmonary function in PH rats.** (**A**) Flowchart of modeling and processing of PH rats. (**B**) Flowchart of body weight changes of rats in each experimental group from the beginning of PH modeling to the end of modeling, and body weight changes of rats were recorded every 6 days. (**C**) Detection of changes in systolic pressure of the right ventricle in each experimental group after the end of modeling of PH rats. (**D**) Detection of changes in Fulton Index in each experimental group. (**E**) Detection of changes in expression of respiratory rate in each experimental group. (**F**) Echocardiography measurement of pulmonary artery (PA) function showing normalization of pulmonary acceleration time (PAT). Changes in the expression of respiratory rate. (**F**) Echocardiography measurement of pulmonary artery (PA) function showing normalization of pulmonary acceleration time (PAT)/pulmonary ejection time (PET) ratio. (**G**) Detection of changes in EF, FS, LVVs, LVVd, LVIDs, and LVIDd in MCT-induced PH rats after 4-HS treatment.

Time flow chart of the *in vivo* experiments is shown in Figure 4A. Firstly, we observed the changes in body weight of rats in each experimental group during the 6 weeks of MCT treatment (*n* = 5), and the statistical results showed that the body weight of rats in the PH group decreased significantly (*P* < 0.0001), whereas the body weight of rats in the 4-HS-treated group increased significantly compared with that of rats in the PH group (*P* < 0.0001), but it was still lower than that of rats in the normal control group as shown in Figure 4B. After this, we detected the changes in the parameters related to cardiopulmonary function of rats. After that, we examined the changes of the parameters related to cardiopulmonary function of rats, and the parameters of Right ventricular Systolic Blood pressure (RVSP) (*P* < 0.0001), Fulton index (*P* < 0.0001), and respiratory rate (*P* = 0.0008) increased significantly in the PH group compared with the control group, while the parameters of 4-HBP were significantly increased by adding 4-HBP (*P* < 0.0001). Significantly increased, while the addition of 4-HS was able to counteract the effects of MCT on RVSP (*P* < 0.0001), Fulton index (*P* = 0.0210), and respiratory rate (*P* = 0.0352) parameters in PH rats as shown in Figure 4C–4E.

In addition, MCT induced a significant decrease in the PAT/PET ratio in the PH rat model group compared with the control group (*P* < 0.0001), whereas 4-HS treatment was able to reverse the change in the PAT/PET ratio as shown in Figure 4F (*P* < 0.0001). In addition, the PH model group showed a significant increase in EF (*P* < 0.0001), FS (*P* = 0.0142) and a significant decrease in LVVs (*P* = 0.0142), LVVVd (*P* = 0.0142), LVIDs (*P* = 0.0142), LVID (*P* = 0.0142), and a significant decrease in LVID (*P* = 0.0142), as compared to the control group, and 4-HS treatment was able to reverse this effect as shown in Figure 4G.

**Figure 5:**
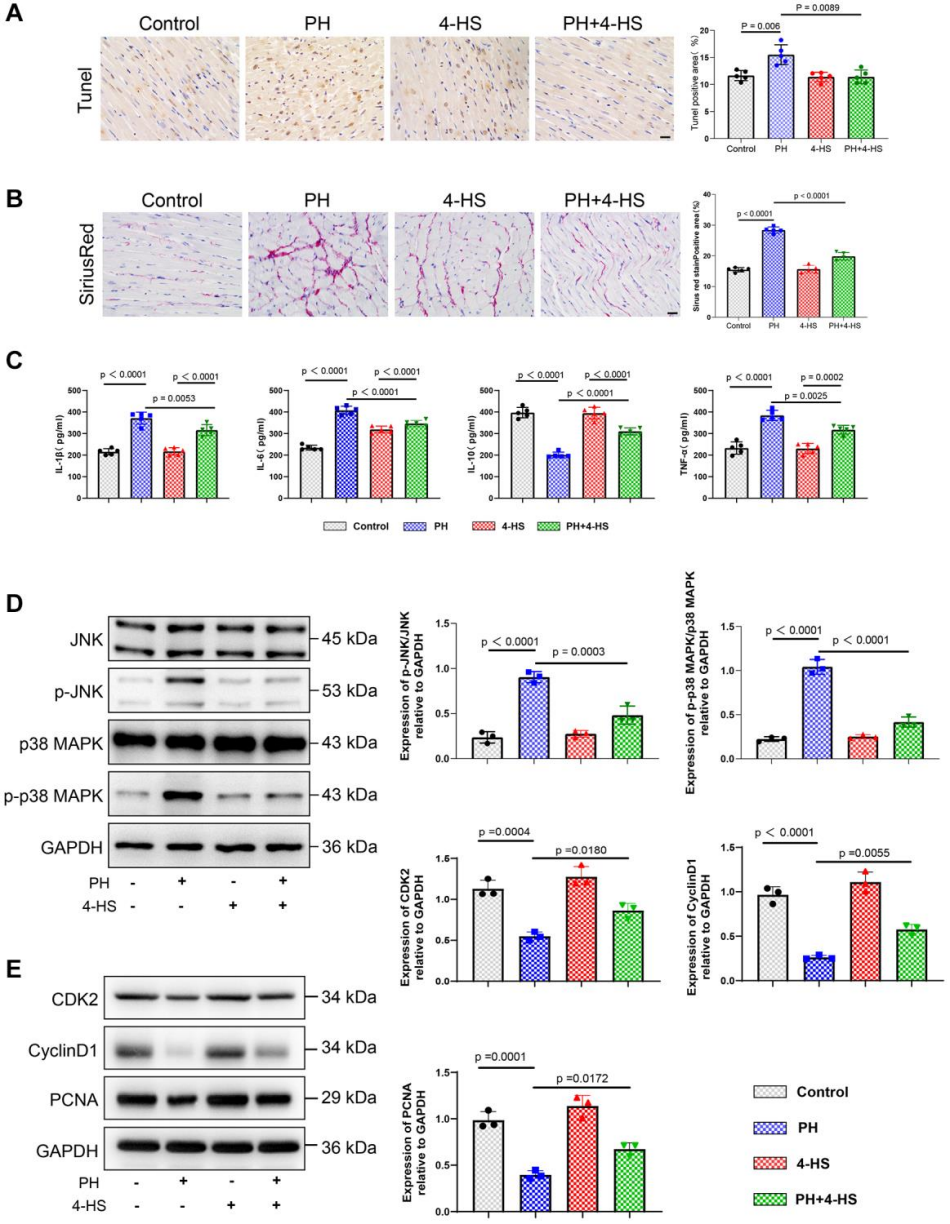
**Protective effect of 4-HS on right ventricular tissue in PH rats.** (**A**) TUNEL staining to detect the effect of 4-HS pretreatment on apoptosis in right ventricular myocardial tissue (Scale bar, 50 μm). (**B**) Sirius scarlet staining to detect the effect of 4-HS pretreatment on right ventricular myocardial tissue (Scale bar, 50 μm). Effect of fibrosis. (**C**) ELISA assay to detect changes in the expression of inflammatory factors IL-1β, IL-6, IL-10, and TNF-α in the serum of rats in each group of the experiment. (**D**) Western blot assay to detect changes in the expression of JNK, p-JNK, p38 MAPK, and p-p38 MAPK proteins in the tissues of pulmonary arteries in rats. (**E**) Western blot assay to detect changes in the expression of CDK2, CyclinD1, and PCNA proteins in the tissues of pulmonary arteries in rats.

TUNEL tissue staining results showed that the apoptosis ratio was significantly increased in the PH group compared with the control group (*P* = 0.006), whereas the apoptosis ratio was significantly decreased in the 4-HS group compared with the PH group (*P* = 0.0089) as shown in Figure 5A. Results of the Sirius scarlet staining experiments expressed a significant thickening of right ventricular myocardial fiber cross-sectional area in the PH group compared with the control group, and a significant decrease in the 4-HS Myocardial fiber tissue was significantly reduced in the 4-HS pretreated group compared with the PH group (*P* < 0.0001) as shown in Figure 5B. ELISA results showed that the expression levels of IL-6 (*P* < 0.0001), TNF-α (*P* < 0.0001), and IL-1β (*P* < 0.0001) were increased in the PH group, whereas IL-10 (*P* < 0.0001) was decreased, and that IL-10 (*P* < 0.0001) was increased in the HS-4 group compared with the PH group (*P* < 0.0001) as shown in Figure 5C. The expression levels of IL-6 (*P* < 0.0001), TNF-α (*P* = 0.0025), and IL-1β (*P* = 0.0053) were decreased, whereas IL-10 (*P* < 0.0001) was increased in the HS-4 group compared with the PH group. We assayed changes in the expression of the JNK/p38 MAPK signaling pathway and proliferation-related metrics in rat myocardial tissues in *in vivo* experiments, and as we expected the results were consistent with the *in vivo* experiments, 4-HS was able to inhibit the activation of the JNK/p38 MAPK signaling pathway and to enhance the expression levels of CDK2, CyclinD1, and PCNA proteins (Figure 5D, 5E).

## DISCUSSION

Natural medicine is an important way to develop low-toxicity and high-efficiency medications, and Sesamin, as a natural plant extract, have been used in asthma [14], colorectalitis [15], cardiovascular disease [16], and many other diseases, etc. Although the pharmacological effects of Sesamin are exciting, they have low solubility and poor water solubility [13], which hinders the efficacy of Sesamin, the laboratory structural modification of Sesamin to enhance their solubility and pharmacological effects, according to the literature, pulmonary arterial hypertension will make the pulmonary artery tissues in a long-term hypoxic or inflammatory environment [17], so we established a cellular model of pulmonary arterial hypertension disease using LPS and a hypoxic environment in PASMC *in vitro*, and we evaluated the anti-inflammatory effects of Sesamin and 4-HS, and as we expected, 4-HS showed better anti-inflammatory effects than Sesamin at the same drug concentration. We also confirmed in *in vitro* experiments that PASMC pretreated with 4-HS were able to counteract the cellular damage brought about by hypoxia induction. It was found that serum IL-6 in PH patients [18], TNF-α [19] and IL-1β [20] levels were significantly higher compared to the normal population and negatively correlated with lung function in these patients, and that these inflammations could drive vascular remodeling and elevated pulmonary arterial pressure [21] and IL-10 is lowly expressed in patients with PH [22]. In addition, the expression of IL-6, TNF-α, IL-1β, and IL10 inflammatory factors correlated with the severity of PHA patients [23]. Our findings that 4-HS was able to downregulate the expression levels of IL-6, TNF-α, and IL-1β and upregulate IL-10 both *in vivo* and *ex vivo* and attenuate the inflammatory response in a pulmonary arterial hypertension disease model are consistent with the anti-inflammatory effects of Sesamin reported in other diseases [24, 25]. In addition, we found that 4-HS pretreatment ameliorated the damaging effects on cardiopulmonary function in the MCT-induced PH model in an *in vivo* rat model of PH disease, and was also able to reduce myocardial tissue apoptosis and fibrosis in rats.

The JNK/p38 MAPK signaling pathway is one of the most critical intracellular pathways, and Wilson et al. found that PASMC cell proliferation was dependent on the activation of JNK and p38 MAPK phosphorylation, and that blocking phosphorylation inhibited PASMC cell proliferation [26] and that regulation of the JNK/p38 MAPK signaling pathway also mediates PH vascular remodeling [27] Therefore, we focused on the JNK/p38 MAPK signaling pathway. In our *ex vivo* and *in vivo* disease models, 4-HS was able to inhibit the expression levels of p-JNK and p-p38 MAPK proteins, which in turn had a protective effect on cardiopulmonary function in rats, and 4-HS was also able to restore the proliferative capacity of PASMC and to reduce inflammatory responses. Sesamin have also been reported in other diseases to inhibit the expression of the JNK/p38 MAPK signaling pathway [28, 29], consistent with the inhibitory effect of 4-HS on JNK/p38 MAPK. Previous studies have shown that CDK2, CyclinD1 and PCNA are all key indicators of cell proliferation [30–32]. *In vitro* cell experiments, we found that 4-HS can reduce the expression levels of CDK2, CyclinD1 and PCNA proteins, and resist the abnormal proliferation of PASMC caused by hypoxia. Interestingly, 4-HS was sufficient to raise expression levels of CDK2, CyclinD1, and PCNA proteins in *in vivo* models. Our results confirm that 4-HS can promote the proliferation of cardiomyocytes, inhibit the abnormal proliferation of PASMC, and play a protective role in right ventricular failure caused by pulmonary hypertension.

In conclusion, the biggest innovation of this study is that it is the first time to point out the protective effect of 4-HS on right ventricular failure due to PH, and it is the first time to find that 4-HS is able to inhibit the activity of the JNK/p38 MAPK signaling pathway, because the disease model of right heart failure due to PH is more complex, the limitation of this paper is that the experimental model *in vitro* cannot completely mimic the real disease situation *in vivo*, although we in *ex vivo* experiments verified that 4-HS was able to inhibit the activity of the JNK/p38 MAPK signaling pathway, but no reverse validation experiments were carried out *in vivo*. Moreover, we did not confirm whether 4-HS can play a protective role in rats *in vivo*, which is also the focus of our future studies. In conclusion, we have discovered a new target drug for the treatment of right ventricular failure due to PH, which will provide a theoretical basis for subsequent researchers.

## CONCLUSIONS

In the present study, we demonstrated for the first time *in vitro* and *in vivo* that 4-HS has a protective effect on cardiopulmonary function in MCT-induced PH rats by reducing tissue inflammatory response and cardiomyocyte apoptosis and may be associated with the inhibition of JNK/p38 MAPK signaling pathway activation. In conclusion, 4-HS is of great developmental value and is expected to be a target drug for the prevention and treatment of diseases associated with right ventricular failure due to PH.
